# Rules of tissue packing involving different cell types: human muscle organization

**DOI:** 10.1038/srep40444

**Published:** 2017-01-10

**Authors:** Daniel Sánchez-Gutiérrez, Aurora Sáez, Pedro Gómez-Gálvez, Carmen Paradas, Luis M. Escudero

**Affiliations:** 1 Departamento de Biología Celular, Universidad de Sevilla and Instituto de Biomedicina de Sevilla (IBiS), Hospital Universitario Virgen del Rocío/CSIC/Universdad de Sevilla, 41013 Seville, Spain; 2 Dpto. Teoría de la Señal y Comunicaciones. Universidad de Sevilla, Cmno, de los descubrimientos s/n, 41092, Sevilla, Spain; 3 Neuromuscular Disorders Unit, Department of Neurology, Instituto de Biomedicina de Sevilla (IBiS), Hospital Universitario Virgen del Rocío/CSIC/Universdad de Sevilla, 41013 Seville, Spain

## Abstract

Natural packed tissues are assembled as tessellations of polygonal cells. These include skeletal muscles and epithelial sheets. Skeletal muscles appear as a mosaic composed of two different types of cells: the “slow” and “fast” fibres. Their relative distribution is important for the muscle function but little is known about how the fibre arrangement is established and maintained. In this work we capture the organizational pattern in two different healthy muscles: biceps brachii and quadriceps. Here we show that the biceps brachii muscle presents a particular arrangement, based on the different sizes of slow and fast fibres. By contrast, in the quadriceps muscle an unbiased distribution exists. Our results indicate that the relative size of each cellular type imposes an intrinsic organization into natural tessellations. These findings establish a new framework for the analysis of any packed tissue where two or more cell types exist.

Cell organization in any given tissue is a highly regulated process that controls major shape changes during morphogenesis and eventually, tissue and organ functionality. In the last decade, many studies have used packed tissues, such as epithelia, as models to understand how cell organization determines the fate of an organ^1^,^2^,^3^,^4^,^5^,^6^,^7^,^8^,^9^. In most of these works, the study of epithelial organization was based on the analysis of the distribution of the cells’ sides, assuming that apical regions of epithelial cells behave as polygons. Skeletal muscle, which is composed of closely arranged fibres separated by a fine layer of connective tissue called the endomisium^10^, is another example of a packed tissue. In any biopsy section, skeletal muscle appears as a mosaic of fibres organized as polygons in a tessellation—an arrangement that leaves no empty space between the individual units. Therefore, skeletal muscle has been used as a model to understand the processes behind the regulation of cell organization^11^,^12^.

In a previous publication, we have introduced *network theory and Centroidal Voronoi tessellations (CVT)* as tools in the study of the organization of packed tissue^12^,^13^. In these reports, specific mathematical concepts were used to establish quantifiable variables that can define the organization of natural packed tissues, such epithelia or skeletal muscle. Our results showed that the use of CVT adds new insightful information, since this method allowed us to infer some biophysical properties from the packed tissues that were also supported by computer simulations.

Packed tissues obey several laws that relate area with organization. These includes *Euler’s Theorem* that states that the average number of neighbours of a cell will be close to six; *Lewis’ law* that linearly relates the average area of a cell with its number of sides (i.e, small cells tend to have fewer sides, and big cells tend to have higher number of sides); and the *Aboav-Weaire law* that establishes an inverse relationship between the average number of sides of a cell and the average number of sides of their neighbours^5^,^8^,^14^,^15^,^16^,^17^,^18^. In addition, it was shown that there is a physical constraint affecting natural packed tissues that restricts them to specific organizations. The distribution of cell sides in these arrangements is similar to the polygon distribution data revealed by the CVT analysis.

All previous studies investigating tissue organization have considered tissues formed by cells with the same properties and capabilities: that is, equivalent entities that could transiently vary their properties depending on the cell cycle stage or changes in the cytoskeleton^12^,^19^,^20^,^21^,^22^,^23^. Here we analysed the organization of skeletal muscle tissues, considering the distribution of myofibres into fast and slow twitch type^24^, which are determined by the specific myosin protein expressed in each fibre. This distribution establishes a mosaic or “checked” pattern that is a characteristic feature of skeletal muscle. The identity of a fibre is determined during development by myogenic factors (prenatal), which will be later modulated by neural and hormonal factors (postnatal)^25^,^26^,^27^. The proportion of fibre type and the size of the fibres can vary between different muscles, species, gender or even individuals, in the case of humans^27^. In different developmental stages and during aging, it is possible to find transitions between slow and fast fibres and vice versa. This, together with that fact that fibre pattern can be remodelled by external factors such exercise, account for the high heterogeneity in the fibre pattern in muscle tissue^27^.

The neuromuscular system is constituted by motor neurons in the spinal cord, the peripheral motor neurons, the neuromuscular junctions, and the muscles themselves. Neuromuscular diseases are a large group of pathologies caused by the alteration of one, or more, of these components, with very heterogeneous etiology and course.

The evaluation of the changes in the morphological characteristics of a given biopsy, with respect to normal muscle, is one of the main features for the diagnosis of a neuromuscular disorder^28^,^29^,^30^,^31^. Morphological pathogenic features evaluated in a muscle biopsy include alterations of fibre size, position of nuclei, and the amount of connective tissue or necrotic fibres. Changes of the distribution pattern of slow and fast fibres can also be detected: a typical feature of the neurogenic disorders such neuropathies or amyotrophic lateral sclerosis^11^,^31^. In addition, a switch from fast to slow twitch type fibre and predominance of one fibre type, or even uniformity of fibre type, are detected in some types of myopathies^32^.

Since the precise way the skeletal muscle degenerates under pathogenic conditions is critical to determine the cause of many neuromuscular disorders, the accurate definition of the features in normal muscles is also essential to better identify the disease. Considering that most of muscle biopsies are taken from biceps brachii and deltoids muscles in upper limbs, and quadriceps, tibialis anterior and gastrocnemius in lower limbs, these are the muscles that should hence be described under normal conditions from a clinical perspective. To analyse the structural and organizational pattern of skeletal muscles, a high number of samples is mandatory^11^,^13^,^33^. Therefore, due to the number of available normal samples and the morphological similarity between them regarding distribution of the type of fibre, we selected biceps brachii and quadriceps muscles for study.

In this work we integrate geometric and topological data to capture an organizational signature in packed tissues with two different cell types. Our results indicate that biceps brachii and quadriceps can be distinguished based the pattern of slow and fast cells. Our data demonstrate that the mosaic defined by these two cell types shows a differential organization for skeletal muscles.

## Results

### Computerized analysis of biceps brachii and quadriceps biopsy images

**Figure 1:**
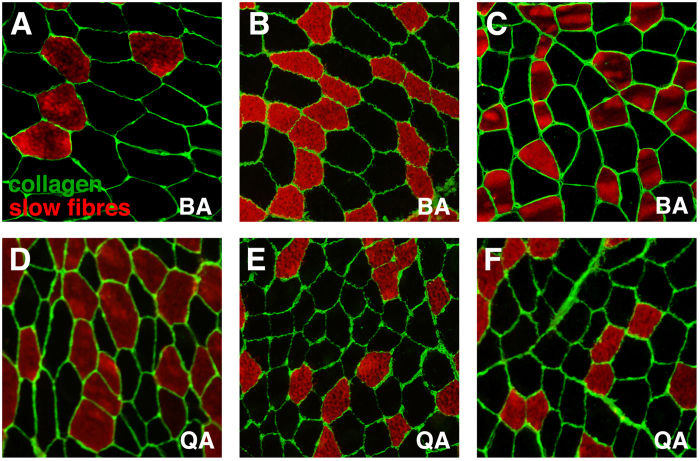
Images from control human muscle biopsies. Fluorescence images corresponding to control biopsies showing collagen VI content including the endomysium and perimysium (green), slow fibres (red) and fast fibres (black). Collagen labels the outline of all the fibres. Fast fibres are identified by the absence of slow myosin heavy chain staining. (**A,B,C**) Images from control biceps brachii. **(E,F,G**) Images from control quadriceps.

We compared biceps brachii (BA) and quadriceps (QA) muscles from control male adult individuals in terms of morphological characteristics of their fibres. Thin sections of biopsies were analyzed using immunohistochemical staining. We combined anti-collagen VI antibody, that provides the outline of the muscle fibres (and enables the quantification of the amount of collagen in the tissue) and anti-myosin slow (type I) specific antibody that allows the identification of fibre type (Fig. 1). In the case of BA, 18 biopsies were analyzed, obtaining 34 micrographs and 90 Region Of Interest (ROI) (Fig. 1A–C and Table S1). 6 QA biopsies were used to obtain 9 micrographs and 25 ROI (Fig. 1D–F and Table S1). Human inspection of the different regions of interest (ROI) is not sufficiently discriminating to extract patterns that enable differentiating both types of muscles (Fig. 1). We therefore used a computerized approach, outlined below, aiming to capture a characteristic signature from each image. First, the images were segmented to identify the outline of the fibres and the collagen content^11^,^33^. Then the values for a series of 14 geometrical characteristics (14 first features in Table 1) and the proportion of slow cells (feature 69 in Table 1) were calculated. In each type of muscle, samples were very heterogeneous and presented a wide range of values for each characteristic. We started examining some of these geometric characteristics, comparing their averages values between both types of muscles. BA fibres were around 33% bigger than QA in terms of average area, average area of slow cells and fast cells, and average major and minor axis (Table S2). Both BA and QA presented a lower proportion of slow fibres (around 31% versus 25% respectively) than fast fibres. Interestingly, in the case of QA, the average area of fast and slow fibres was virtually the same; meanwhile, in the case of BA the average size was bigger in the fast fibres compared with the slow fibres (Table S2).

### Biceps brachii and Quadriceps present different organization of fibres

**Figure 2:**
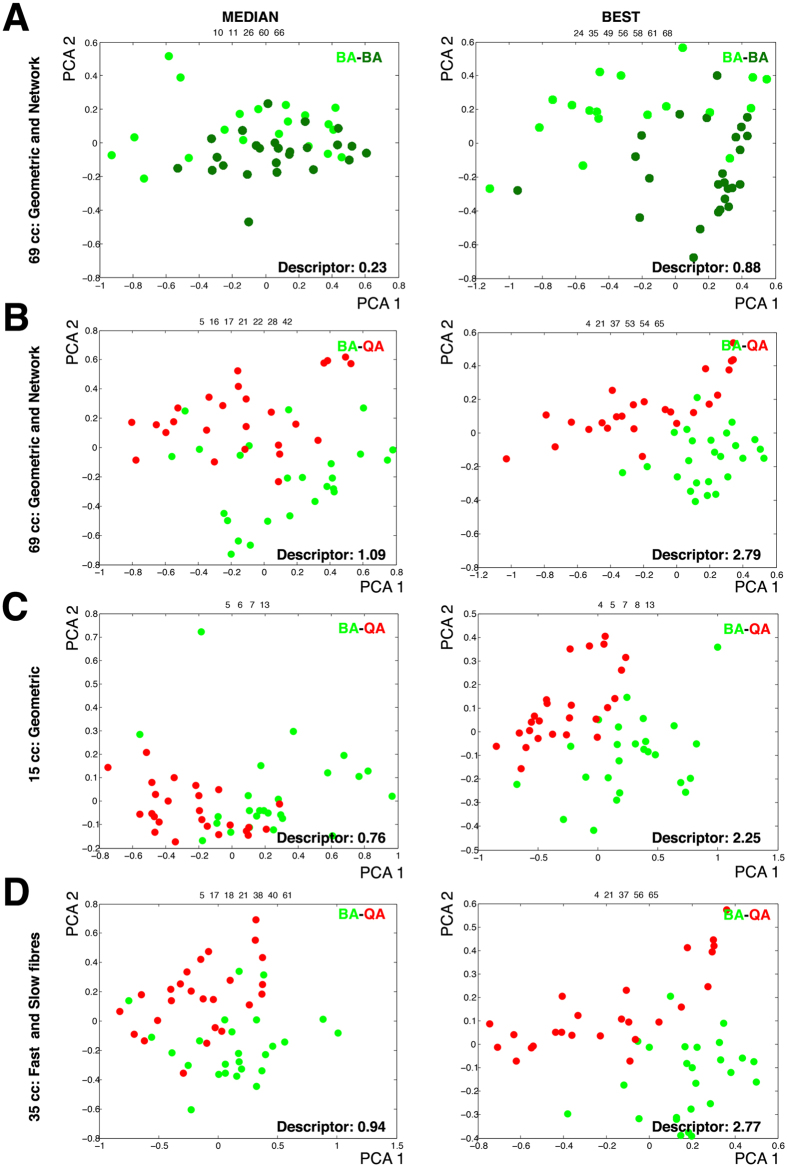
Principal component analysis graphs for different combinations of muscle type images and characteristics. We have selected the PCA graphs corresponding to the comparison that provide the “median value” (left panels) and the “best value” (right panels) as representatives of the whole range of 1,000 comparisons performed. Representative PCA graphs for the comparisons of two groups of 25 images. After calculate the PCA descriptors for the 1,000 random comparisons the PCA graphs corresponding to the comparisons that provide the “median value” (left) and the “best value” (right) are shown. The green dots (dark or light) represent BA images. The red dots represent QA images. The numbers over the graphs indicate the selected characteristics. (**A)** 25 images randomly taken from a set of 90 samples of BA vs other different 25 images using the set of 69 cc. (**B)** 25 images randomly taken from a set of 90 samples of BA versus 25 QA images using the set of 69 cc. (**C)** 25 images randomly taken from a set of 90 samples of BA versus QA images using the set of 15 cc (14 geometric and the proportion of slow cells). (**D)** 25 images randomly taken from a set of 90 samples of BA versus QA images using the set of 35 cc.

We compared the organization of BA and QA samples using a network approach that evaluates topological characteristics, aiming to identify small organizational differences between apparently similar images^11^,^34^. The method is based in the consideration of the tissue as a network of cell to cell contacts^13^. Under this premise, we extracted the values for 54 “network” characteristics (features 15-68 in Table 1) besides the 14 geometric features and the proportion of slow cells. In this way, we obtained a vector of 69 features for each muscle ROI. Due to the large difference in the number of ROIs (90 BA vs. 25 QA) we designed a protocol to use the whole data and at the same time be able to obtain comparable results. The protocol consisted of performing 1,000 combinations of ROIs. Each combination was done using 25 images of each group. To obtain a baseline for our evaluation system, we first performed 1,000 comparisons using only BA images: two groups of 25 BA images were chosen randomly from the total 90 each time. Each comparison was used to perform a feature selection step that chose the most relevant characteristics from the totality of features assayed. These selected features were used to perform a Principal Component Analysis (PCA) and obtain a value for the “PCA descriptor” that quantified the degree of separation between both groups of images^34^ (and Methods). The values of the PCA descriptor ranged from 0.08 to 0.88, and presented a median value of 0.23. We selected the PCA graphs corresponding to the comparison that provides the “median value” and the “best value” of PCA descriptors as representatives of the whole range of 1,000 comparisons performed (left and right panels respectively in Fig. 2A–D). We then performed another 1,000 randomizations. In each of these randomizations, 25 images from the 90 BA were selected and compared with the 25 QA images. Using this approach, the values of the PCA descriptor ranged from 0.49 to 2.79, with a median value of 1.09 (Fig. 2B). The comparison of the PCA graphs corresponding to the median and best values in each case suggested that the separation was largely improved in the case of BA-QA with respect to BA-BA (Fig. 2A,B). We also observed that the BA-QA values were lower when using the set of 15 characteristics (14 geometric features and the proportion of slow cells; ranging from 0.25 to 2.25, with a median value of 0.76, (Fig. 2C), indicating the importance of the network characteristics to improve the separation. This trait was also illustrated when comparing all 90 ROI from BA with the 25 QA samples. The use of the network characteristics improved the separation, although in these cases the differences were smaller due to the imbalance of sample numbers between BA and QA (Fig. S1A–C).

### Similar muscles differ in the organization of fast and slow fibres

We examined the features that were relevant to distinguish BA versus QA samples, trying to understand the biological differences between these two seemingly similar muscles. Each feature selection step selects a maximum of 7 features per comparison. We calculated the rate of appearance of each feature in each one of the 1,000 comparisons performed in each case. In our baseline assay, the 1,000 BA-BA comparisons, we did not find clear predominant characteristics. In this case, the most frequent characteristic appeared only in 20.6% of the randomizations (Table 2, features above the 15% of frequency). We compared these results with the BA-QA assay. In this case there was a clear predominance of some characteristics over others (Table 2, features above the 25% of frequency). This result indicated that different combinations of BA images could be separated from QA images using the same features. In short, these results suggest the existence of some general differences between BA and QA. The most frequent characteristics appearing in the BA-QA comparisons were mainly related to the geometry or organization of the different types of fibres (the nine most frequent features in Table S3). In particular, the “standard deviation of the area of the slow cells” and the “number of slow neighbours of fast cells” were the two most relevant features. This suggested that the difference between BA and QA could stem from the distribution of fast and slow fibres. To test this idea, we repeated the 1,000 BA-QA comparisons using only the 35 characteristics that were specifically related to fast and slow fibres. The distribution of values for the PCA descriptor was still high (ranging from 0.35 to 2.77, with a median value of 0.94, Fig. 2D). We also observed a predominance of the same type of features than in the previous experiment using 69 characteristics (Table S3).

### Importance of the proportion of fast and slow cells for muscle organization

**Figure 3:**
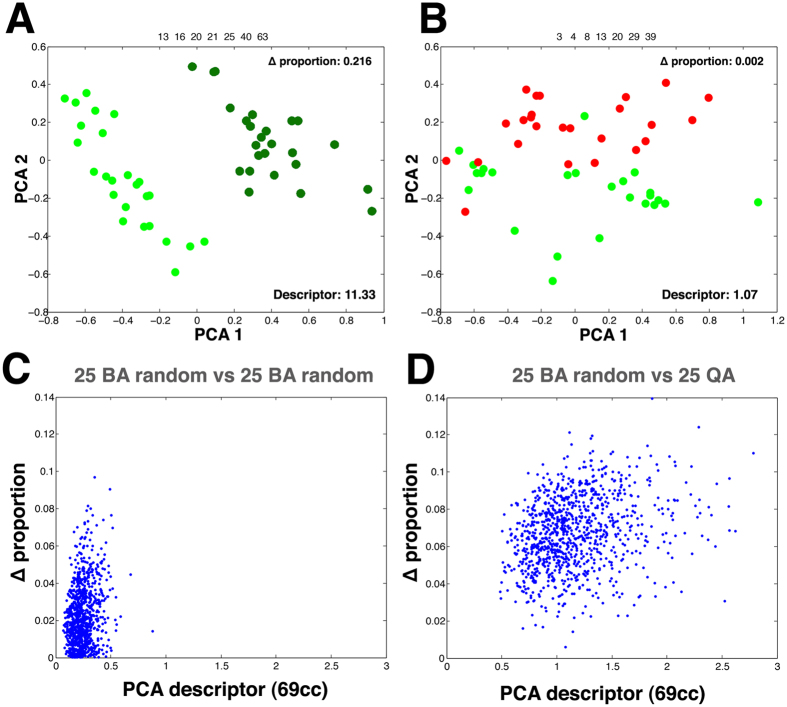
Influence of the proportion of slow fibres in the muscle organization. (**A**) Comparison of 25 images from BA (light green dots) vs 25 images from BA (dark green dots) using two groups of BA images with a very different percentage of slow fibres (Δ proportion = 0.216) and a set of 69 characteristics. The result is a clear separation of both groups with a PCA descriptor of 11.33. (**B**) Comparison of 25 images from BA (green dots) with very similar percentage of slow fibres (Δ proportion = 0.002) than the 25 QA images (red dots) and a set of 69 characteristics. The graph shows some overlap between the two groups (PCA descriptors = 1.07). (**C)** Graph representing the 1,000 random comparison of 25 images random from BA versus 25 images random from BA (blue dots). “Δ proportion” of slow fibres is represented against the PCA descriptor value of the same random comparison. (**D)** Graph representing the 1,000 random comparison of 25 images random from BA versus QA (blue dots). “Δ proportion” of slow fibres is represented against the PCA descriptor value of the same random combination.

We show here that BA and QA present different average proportions of fast and slow fibres (Table S2). This trait influences the values of the network characteristics related to slow and fast fibres. We tried to evaluate the importance of these relative differences for muscle organization. To do that, we selected two groups of 25 BA images with very different percentages of slow fibres. Using 69 characteristics for the comparison, the PCA graph showed two clearly separated groups, and the PCA descriptor value was extremely high: 11.33 (Fig. 3A). In this case, the difference between the average percentages of slow cells between these two groups was 0.216 (we will call this value Δ proportion). In parallel, we compared QA samples with a selection of BA samples with the percentage of slow cells more similar to QA (a Δ proportion value of 0.002). In this case, there was some degree of separation with a descriptor of 1.07 when using 69 characteristics (Fig. 3B and Table 3). Interestingly, this value was very similar to the median value of the 1,000 BA-QA comparisons (1.09; Fig. 2B). To further investigate the relation between Δ proportion and the separation of the groups of images, we used 1,000 BA-BA comparisons to plot the values for the PCA descriptor against its corresponding Δ proportion values (Fig. 3C). We observed a poor association between the increase of the Δ proportion and the PCA descriptor (Pearson´s coefficient r = 0.2435). Likewise, we did not find a significant correlation when we used the 1,000 BA-QA comparisons (Fig. 3D, Pearson´s coefficient r = 0.2735). These results suggested that the proportion of slow cells is not the main factor responsible for the differences between BA and QA tissues.

### The relative size of slow and fast fibres affects their relative distribution

**Figure 4:**
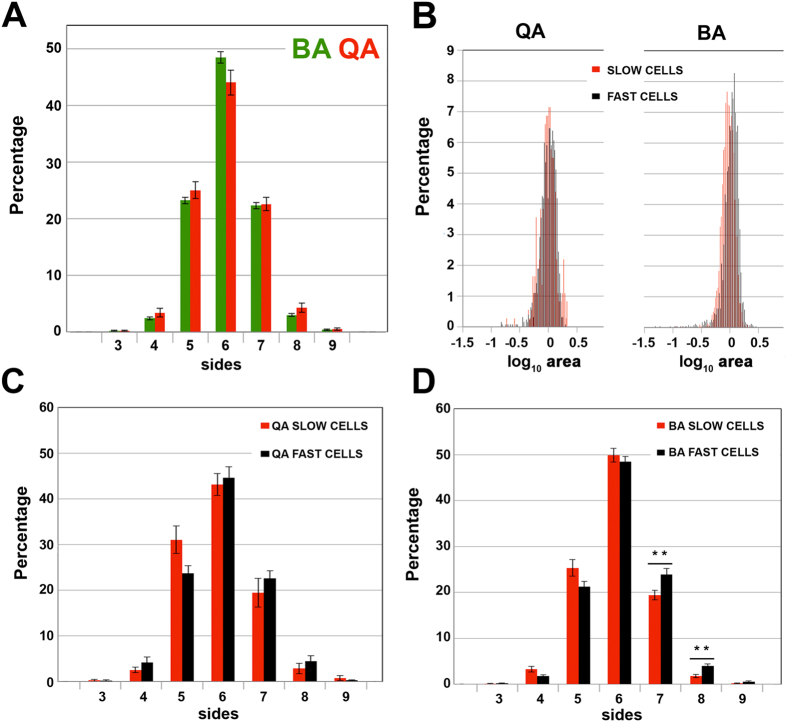
BA and QA present differences in polygon and area distribution of their slow and fast fibres. (**A**) Polygon distribution of BA fast fibres (black) and BA slow fibres (red). The error bars represent the standard error. (**B**) Comparison of the area distribution of QA fast and slow cells (left panel) and the area distribution of BA fast and slow cells (right panel). (**C**) Polygon distribution of QA fast and slow fibres. (**D**) Polygon distribution of BA fast and slow fibres. The frequency of each type of polygon in both sets of images is represented. The error bars represent the standard error. **p value < 0.001, Wilcoxon test (See also, Table S4).

The muscles fibres are arranged in bundles. Moreover, the analysis of muscle tissue sections revealed a significant similarity to tessellations of convex polygons. This feature has been previously used to try to capture the organization of packed tissues^4^,^12^,^23^,^35^. Based on this trait, we examined our biceps brachii and quadriceps samples, and found that they presented a similar polygon distribution (Fig. 4A and Table S4; MANOVA *p value* = 0.3196). In packed cellular arrangements, the area and the number of neighbours are related, following Euler´s theorem and Lewis and Aboav-Weaire laws^5^,^8^,^14^,^15^,^16^,^17^,^18^. As mentioned above, one of the obvious differences between BA and QA samples is the average relative size between fast and slow fibres. We examined whether this disparity was extended to the distribution of fibre size (Fig. 4B). In the case of QA, the distribution of fast fibre area and the distribution of slow fibre area presented a very high level of overlap (Fig. 4B left panel). In contrast, BA distributions of slow and fast cell areas were slightly displaced, since a substantial proportion of slow cells was smaller than the fast cells (Fig. 4B right panel). Although in both cases we were not able to find significant differences between slow and fast fibre area distribution (Kolmogorov-Smirnov test; QA: *p value* = 1; BA: *p value* = 0.3309) we decided to continue the analysis on the relation between area distribution and organization. Following the principles of the Lewis and Aboav-Weaire laws, the small difference in area distribution of slow and fast cells in BA could bias their organization: bigger cells (fast) should tend to have a higher number of neighbours, and these neighbours should tend to be smaller cells with a lower number of sides (slow). Therefore, we analyzed the polygon distribution of both types of fibres in the QA and BA images (Fig. 4C,D and Table S4). Using the MANOVA test to compare slow and fast polygon distributions, we were not able to find significant differences in the case of QA (Fig. 4C, MANOVA *p value* = 0.1434). Conversely, BA samples presented distributions significantly different (Fig. 4D, MANOVA *p value* = 0.0037). In addition, we statistically compared the frequency of each polygon class between slow and fast fibres (Methods). Again, there were no differences in the case of QA (Table S4). In BA, we found that the number of slow fibres that were heptagons and octagons was significantly lower than among fast fibres (Fig. 4D and Table S4). Based on these results, we propose that the small differences in the area distribution found in the BA samples imposed a degree of order in the BA organization that it is absent in QA.

### Slow and fast fibres present an intrinsic organization in the biceps brachii

**Figure 5:**
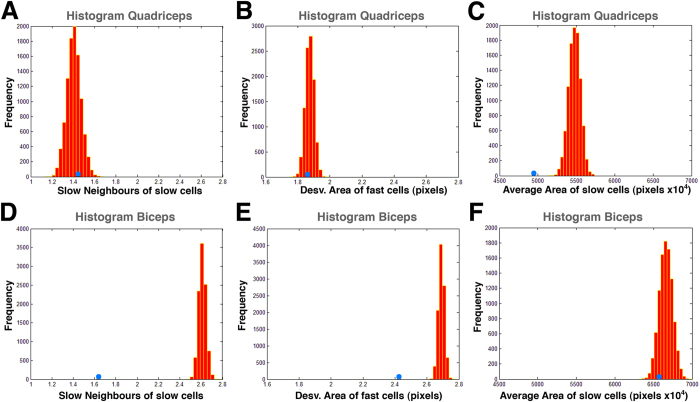
Frequency of values for characteristics depending of the distribution of fast and slow fibres. The histograms show the frequency of values for several characteristics related to the distribution of fibres type from 10,000 randomizations of the fast and slow fibres. Blue circles show the value of the characteristic for the real distribution of fast and slow cells in the muscle. (**A**,**D)** Histogram for the characteristic “slow neighbours of slow cells” in QA and BA respectively. The real value is similar to the median of random values. (**B**,**E)** Histogram for the characteristic “deviation area of fast cells in QA and BA respectively. (**C**,**F)** Histogram for the characteristic “average area of slow cells” in QA and BA respectively. In the cases (**C**,**D**,**E)** the real value is lower than the random values.

Our data suggested that BA and QA samples presented differences related to the organization of their two types of fibres. To test this hypothesis, we performed simulations where in each ROI, every cell was designed fast or slow randomly (while maintaining a constant percentage of fast and slow fibres). Plausibly, this approach changed the values for the 34 characteristics specifically related to fast and slow fibres properties. We obtained the average value for each characteristic considering all the images of each category (90 ROI in the case of BA and 25 for QA). Then we plotted the distribution of the values for each characteristic and compared them with the distribution of values for 10,000 randomizations of fibre type (Fig. 5A–F and Table 3). We expected that if a characteristic was not affected by the fibre-type randomization, the real value would fall inside of the distribution of random values. This was the case for all the features, except for two, when analyzing QA samples (Fig. 5A–C and Table 3). In contrast, more than a half of BA characteristics presented the real value displaced from the distribution of random data (Fig. 5D–E and Table 3). In some cases, the real value was very different from the randomized. For example, the real average number of “slow neighbours of slow cells” was clearly lower than any of the randomized data (Fig. 5D). This suggested that slow cells in the BA muscle were mainly surrounded by fast cells and not by other slow cells (i.e. slow fibres tended to appear isolated and the randomization grouped them). This result supported that BA organization of fast and slow fibres was not arbitrary.

## Discussion

### Biceps brachii and quadriceps are different in terms of the organization of slow and fast fibres

In this study we integrate and quantify information from two large sets of images from two healthy muscles. Although after visual inspection both sets of images were highly similar (Fig. 1), our computerized analysis revealed a wide heterogeneity between samples from the same type of muscle^27^. For example, the “average area” of quadriceps fibres is bigger than the “average area” of biceps brachii fibres. In contrast, a high proportion of biceps brachii images present fibres bigger than quadriceps fibres (Table S1 and Table S2). Here, we tackled this problem using several approaches that try to incorporate all data from the two sets of images. The first step was to design a protocol to evaluate all the images available (90 BA and 25 QA). Our method allowed us to obtain 1,000 values for the PCA descriptor for each comparison of BA and QA data, and to analyse the differences, or similarities, among all the samples. Our first conclusion is that our method is not able to completely separate both types of images. The representative graphs of the median values of the PCA descriptor show how some BA images are very similar to the QA (Fig. 2B,D left panels). Even the best combinations still present some overlapping of images in the PCA graph (Fig. 2B,D rigth panels). Nevertheless, we have been able to extract some useful information from these type of assays: i) topological characteristics improve the separation of the BA and QA images; ii) the characteristics related to the fast and slow fibres contain most of the relevant information to distinguish BA and QA; and iii) the comparisons using only BA samples (that generated very low descriptor values) serve as a baseline to indicate that the partial separation obtained between BA and QA reflects some general differences between these two types of muscles.

We also analyzed the most frequent characteristics in Table 2, trying to understand which trait is based on disparities in the organization between QA and BA. Interestingly, the six most frequent characteristics of the 1,000 BA-QA comparisons (all appearing in more than the 25% of the cases) are features that were also highlighted in the slow/fast cell randomization assay. This result suggests that the feature selection method considers the characteristics that capture the slow/fast mosaic as the most relevant to distinguish BA and QA organization. The most frequent characteristic is the “S. D. Area of slow cells” indicating the high relevance of the homogeneity in sizes for the slow cells in BA, in contrast to the wider range of sizes in the case of QA (Table S3). The second (appearing in almost half of the cases) is “slow Neighbours of fast cells”. This characteristic would reflect the combination of the difference in the percentage of the slow cells between BA and QA, together with the particular arrangement of slow and fast cells in the BA tissue. The third and fourth characteristics are the Average Strengths of fast and slow cells respectively. These two characteristics combine the information about the size and the number of neighbours of each type of cell. This trait seems slightly more relevant than the “average area of fast cells” (the fifth characteristic) to distinguish between both types of muscles. Finally, we find “S.D. Neighbours of slow cells” indeed reflect the fact that slow cells in BA are less variable, due to their more constant size. Using our method, we have been able to compile all these characteristics to discriminate between both types of samples in the majority of combinations studied. Thus, despite the large heterogeneity among the samples under analysis, we are able to conclude that the distribution of the slow and fast cell types is relevant to differentiate BA and QA images.

### Biceps brachii present a distinct organization derived from the smaller size of the slow fibres with respect the fast fibres

**Figure 6:**
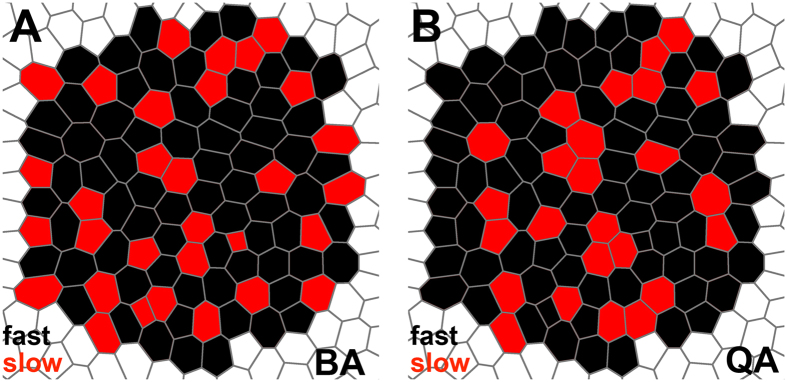
Scheme reflecting the different organization of BA and QA. Slow fibres are labelled in red and fast fibres in black. (**A)** In the BA a high tendency for slow cells to be isolated govern the organization of the tissue. This induces a homogenous distribution of both types of fibres. (**B)** in QA, there is no clear tendency in the organization. Slow fibres can appear isolated or grouped. The distribution is random.

We have explored the possible influence of the distribution of the slow and fast fibres in the global organization of BA and QA tissues. First, we have evaluated the importance of the percentage of each type of fibre (Δ proportion) in the organization of the tissue. We observe that driving this characteristic to a limit, by choosing two sets of images from BA with a very diverse Δ proportion, we are able to obtain a clear separation in the PCA graph (Fig. 3A). However, the values for the PCA descriptor in the 1,000 combinations of BA-BA and QA-BA are clearly lower and do not correlate with the “Δ proportion” (Fig. 3C,D). We believe that this latter result is biologically relevant. It is clear that an abnormally high value for the “Δ proportion” of both sets will impact on all the characteristics analysed. Nevertheless, the 1,000 combinations performed in this study reflect the heterogeneity that can be found in normal muscles among different individuals. Interestingly, our analysis of the “Δ proportion” values shows that a QA-BA comparison with very low “Δ proportion” can still present some differences as in the case shown in Fig. 3B. These data strongly support that other factors, in addition to the percentage of fibres, are playing a role in the organization of muscle tissue. To identify these factors, we analysed the muscle images as an arrangement of convex polygons. In these natural tessellations, the area of the cells and their polygon sides are related in a way that affects the whole organization of the tissue^12^. The distribution of slow and fast fibres areas is slightly different in the case of BA (Fig. 4B). We hypothesize that the reduced size of a large proportion of the slow cells in BA affects the polygon distribution of each type of fibre. This hypothesis is supported by the significant difference (MANOVA test) in polygon distribution between the slow and fast fibres in biceps brachii. The changes are particularly clear in the case of the increment of heptagons and octagons in the subpopulation of fast fibres (Fig. 4D). These heptagonal and octagonal fibres only account for around 20% and 5%, respectively, of the total. However, increasing them results in a reduction of the percentages of the other polygon types (Fig. 4D). In general, in BA there is a higher proportion of slow fibres with a low number of neighbours. In a packed tissue these smaller fibres tend to contact fast fibres with a larger area (according to the Lewis law^8^). Following this argument, for example, a characteristic such “fast neighbours of slow cells” should have a bigger value than the random distribution. This is the case (Table 3). Therefore, we propose that in BA samples, the differences in area and polygon distribution of fast and slow fibres are sufficient to bias the organization of the whole tissue in terms of the arrangement of both types of fibres. On the other hand, QA does not present significant differences between slow and fast fibre polygon distributions, suggesting that for QA, fibre type does not bias the organization of quadriceps. To confirm this idea and further investigate the existence of organizational differences between QA and BA, we used a computational simulation (Table 3 and Fig. 5). For each image, we obtained 10,000 variations where the distribution of the slow and fast fibres was random. In this way we have been able to compare the real values for the 34 characteristics that are related with the distribution of the type of fibres (Table 1) with 10,000 random values. We consider that this is a very robust baseline to compare with, under the assumption that if an inherent organization of slow and fast fibres does not exist in the real tissue, the randomization should not affect these values. This is the case for QA, where only two of the 34 characteristics presented a value out of the range obtained with the 10,000 randomizations (Table 3). Conversely, in the BA experiment, almost half of the characteristics dramatically changed when compared with the real values. We conclude that there is a particular intrinsic arrangement in BA, and that the randomization largely alters this predetermined order. The analysis of the features that deviates from random, together with the integration of the whole set of data extracted, reveal the basis of the biceps brachii organization. We propose that the difference in the size of slow and fast fibres imposes the observed differential polygon distribution between both types of cells. The analysis of the characteristics that differ in BA compared to random provides information to establish a model of how fibres organize in BA muscle (Fig. 6A). We propose that there is a tendency towards isolated slow fibres (small with low number of neighbours) in biceps brachii. This event will affect the whole organization of the tissue, conferring a degree of homogeneity in the distribution of both types of fibre. As a result, there will not be large regions occupied only by fast fibres. This differs from what happen in the “schematic” QA muscle (Fig. 6B), where some slow fibres are isolated and others are grouped without any obvious organizational pattern. For this reason, the randomization assay generates values for most of the characteristics that are in the same range as the real QA values.

In summary, we describe an organizational characteristic pattern based on the differential size of two different types of cells. Although a high heterogeneity exists among the analyzed samples, our systems biology methods have been able to detect a signature that generally distinguishes the biceps brachii from the quadriceps muscles. This discrimination is based on their slow/fast fibre organization. Our results clearly indicate that the relatively larger size of fast fibres in the biceps brachii imposes an intrinsic order that enforces the homogenous distribution of slow fibres in the tissue. On the other hand, there is no bias in the arrangement of both types of fibres in quadriceps.

### Possible applications in biomedicine and other contexts

These results are relevant from a translational point of view. A wide range of pathogenic changes have been described in the skeletal muscle of patients suffering from different neuromuscular diseases, both neurogenic and myopathic disorders. Subtle differences in the response to a pathogenic condition from one muscle to another, could improve the diagnosis in early stages of the disease, which is the goal for any therapeutic intervention in this group of disorders^36^,^37^,^38^. Our results pave the way for the identification of early changes associated with the fibre type distribution in the context of the pathogenesis, which would improve early diagnosis and therapeutic intervention before muscle degeneration.

Muscles are not the only packed tissues where more than one cell-type can be found. During morphogenesis, epithelial cells differentiate into precursors that are maintained within the epithelium for some time. This is the case of the neural crest of vertebrates^39^ or the *Drosophila* sensory organs called mother cells^40^. In an even more complex scenario, in some adult tissues such the *Drosophila* midgut, enteroblasts, stem cells, enterocytes and enteroendocrine cells are integrated in the same layer^41^. In all these examples the relative organization of the different cells types is highly relevant for their function. Here we have described a new framework that can be used to analyze complex packed tissues where epithelial cells start to differentiate, and more than one cell type is founded.

## Methods

### Tissue sampling and histology

For the retrospective analysis of control male muscle tissue, we obtained images from processed biopsies stored in tissue banks at the Virgen del Rocío University Hospital (Seville). All biopsies were performed under informed consent using a standardized protocol^31^ and were processed as described^11^. Fluorescence microscopy was used to detect the outline of the muscle fibres (collagen) and the type (slow myosin heavy chain). The fast fibres were identified by absence of slow myosin heavy chain. The following antibodies were used: mouse anti-myosin heavy chain (slow) (Leica, Newcastle, United Kingdom, clone WB-MHCs; 1:200), and rabbit anti-collagen type VI (Millipore, Temecula, CA, USA, lot number: NG18332|0;1:300). Our database consists of 90 ROI extracted from 34 images which were selected from 14 biopsies for biceps brachii Adult (BA) and 25 ROI extracted from 9 images which were selected from 6 biopsies for quadriceps adult (QA). We selected a ROI with resolution 1,000 × 1,000 pixels from images of 3,072 × 4,080 pixels. In this way it is possible to avoid small artefacts due to the manipulation and staining of the samples.

### Geometric and network feature extraction

Geometric features such as the fibre area or the length of the major and minor axes of the fibre can be extracted from the detected contours. A network of fibre-to-fibre contacts was derived from the segmented image following the steps described in^11^. This allowed to obtain other parameters that take into account the neighbouring vicinity of each fibre, such as the ratio between the fibre area and adjacent fibre areas, or the ratio between the fibre area and the area resulting from the expansion of its contour (computed in the previous step). Finally, features extracted from graph theory applied to the muscle network were also computed (values for all characteristics in each image in Table S1).

In this work a total of 69 characteristics have been computed. They included 14 geometric features, 20 features derived from the muscle network, 34 from graph theory and 1 last characteristic which gave us the proportion of slow cells (Table 1). We defined 3 subsets of characteristics in order to employ it in different comparisons. The first set was performed by all 69 characteristics computed. The second set was defined by 35 characteristics related to slow and fast cell information (in bold in Table 1). The third set was composed exclusively of 14 geometric characteristics (14 first features in Table 1) and the proportion of slow cells.

### Principal Component Analysis features selection

A feature selection step was performed to analyze the discrimination power of a set of characteristics mentioned above that distinguish better two groups of images. The method selects and evaluates features using Principal Component Analysis (PCA) and PCA’s descriptor that quantify the degree of separation between the two groups of images that are compared^34^. We have tested every possible combination of three features in the first iteration and applied the PCA. The method keeps the ten combinations of three features with higher PCA descriptor value. In the second iteration, all features are individually tested again in combination with the ten trios of features. Again, all the combinations are evaluated and the program keeps the five with higher PCA descriptor value for each one of the ten trios. Therefore, at this step the program handles 50 quartets of features. In the next iteration, the same process is repeated but only two best features are added, accumulating 100 quintets of features. The process continues adding only one feature per iteration step. The iteration process is stopped when seven features have been selected or when the value for the PCA descriptor is lower than in the previous step. Finally, we chose the ensemble of features that presented the highest value for the PCA descriptor among the 100 groups.

### Comparison of BA and QA images

Due to the large difference in the number of ROIs (90 BA vs. 25 QA) we designed a protocol to use all the available samples and, at the same time, be able to obtain comparable results. We employed a random process of sample selection to be able to compare the same number of images each time. We selected “25” random ROIs (the smallest quantity of ROIs in one of the groups) to perform the PCA features selection described above. To be sure that we used all the available samples we carried out this process 1,000 times to perform 1,000 comparisons. Therefore, for each comparison, we also obtained 1,000 PCA descriptors and 1,000 sets of relevant characteristics. In order to know which characteristics were most relevant to discriminate two categories along using all the available images, we calculated the rate of appearance of each feature between the selected ones. Table 2 and Table S3 show the most frequent characteristic in each comparison performed in this study.

### Relation between discrimination power and slow fibre proportion

To test if there is a correlation between the values of the PCA descriptors obtained with the 1,000 comparisons and their proportion of fast and slow fibres, we defined the value “Δ proportion” per each one of these 1,000 comparisons. “Δ proportion” was calculated as the difference between the average percentages of slow cells between two groups analyzed in each one of 1,000 comparisons. The Pearson´s correlation coefficient was obtained to analyse the possible correlation between the value of the PCA descriptor and the slow fibre proportion.

### Statistical differences between BA and QA fibre characteristics

We used Multivariate Analysis of Variance (MANOVA) test to perform three comparisons of the polygonal distributions: a) BA total fibres vs QA total fibres, b) BA fast fibres vs BA slow fibres, c) QA fast fibres vs QA slow fibres (Table S4). If *p-value* < 0.05, distributions were considered to be significantly different. The MANOVA tests were performed using only the values for cells with 4, 5, 6, 7 and 8 sides. We discarded the cells with 3, 9 and 10 sides, since they were not present in all the images. In the three comparisons above we also analyzed the differences between the values for each type of polygon. First, we evaluated if the two compared categories values presented similar distribution and variance using Kolmogorov-Smirnov and F-Snedercor tests respectively. In case that data presented different distribution and a different variance, we employed Wilcoxon test to compare the means from both groups. We employed a two tail Student’s t-test to compare the means in the cases where both distribution and variance of the two sets of data were similar (Table S4).

We used the two samples Kolmogorov-Smirnov test to compare “log_10_ Normalized Area” distribution of each category of “BA fast fibres vs BA slow fibres” and “QA fast fibres vs QA slow fibres”.

### Slow and fast cell randomization

In order to know how the spatial distribution of slow and fast cells affected the organization of the muscle, we randomized the positions of fast and slow cells without altering their proportion. In each ROI, every cell was labelled as “fast” or “slow” randomly, maintaining the relative number of fast and slow cells. This process changed the values for the 34 characteristics related to fast and slow properties. We performed 10,000 randomizations for each ROI. For each category and randomization, we calculated the average value of each one of the 34 characteristics. To obtain the “original” value for each characteristic we averaged the values of all the available images (90 for BA and 25 for QA). We plotted the distribution of 10,000 values for each characteristic and compared its minimum, maximum, and median values with the “original” average value of slow and fast cells. (Fig. 5 and Table 3)

### Polygon and area distribution calculations

We analyzed polygon and area distribution in our images to investigate the organization of fast and slow cells in relation to their size (Fig. 4). To make the polygon distribution graphs with the corresponding error bars for each category (BA, BA slow cells, BA fast cells, QA, QA slow cells and QA fast cells) cells were grouped by biopsy.

To compare Area from different categories, we calculated the Normalized Area:


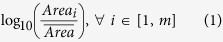


where m is the number of cells in the image, 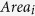 is the size of the cell and 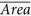 is the mean Area of all the valid cells. We classified the values in bins of 0.02 units to visualize the Normalized Area distribution. The use of the log10 makes the values distribute similar to a normal distribution facilitating the comparison.

## Additional Information

**How to cite this article**: Sánchez-Gutiérrez, D. *et al*. Rules of tissue packing involving different cell types: human muscle organization. *Sci. Rep.* **7**, 40444; doi: 10.1038/srep40444 (2017).

**Publisher's note:** Springer Nature remains neutral with regard to jurisdictional claims in published maps and institutional affiliations.

## Supplementary Material

Supplementary Information

Supplementary Table S1

Supplementary Table S2

Supplementary Table S3

Supplementary Table S4

## Figures and Tables

**Table 1:** List of characteristics analyzed in this study.

Characteristics			
cc	Name	cc	Name
**1**	Average Area	35	Average Strengths
**2**	S. D. Area	36	S. D. Strengths
3	**Average Area of slow cells**	**37**	**Average Strengths of fast cells**
4	**S. D.** **Area of slow cells**	**38**	**S. D.** **Strengths of fast cells**
5	**Average Area of fast cells**	**39**	**Average Strengths of slow cells**
6	**S. D.** **Area of fast cells**	**40**	**S. D.** **Strengths of slow cells**
**7**	Average major Axis	41	Average Clustering Coefficient
**8**	Average minor Axis	42	S. D. Clustering Coefficient
**9**	Average Relation Axis	**43**	**Average Clustering Coefficient of fast cells**
**10**	S. D. Relation Axis	**44**	**S. D.** **Clustering Coefficient of fast cells**
**11**	Average Convex Hull	**45**	**Average Clustering Coefficient of slow cells**
**12**	S. D. Convex Hull	**46**	**S. D.** **Clustering Coefficient of slow cells**
**13**	Average Relation A1/A2	47	Average Eccentricity
**14**	S. D. Relation A1/A2	48	S. D. Eccentricity
**15**	Average Neighbours	**49**	**Average Eccentricity of fast cells**
**16**	S. D. Neighbours	**50**	**S. D.** **Eccentricity of fast cells**
17	**S. D.** **Neighbours of slow cells**	**51**	**Average Eccentricity of slow cells**
18	**S. D.** **Neighbours of fast cells**	**52**	**S. D.** **Eccentricity of slow cells**
19	**slow Neighbours of slow cells**	53	Average Betweenness Centrality
20	**fast Neighbours of slow cells**	54	S. D. Betweenness Centrality
21	**slow Neighbours of fast cells**	**55**	**Average Betweenness Centrality of fast cells**
22	**fast Neighbours of fast cells**	**56**	**S. D.** **Betweenness Centrality of fast cells**
**23**	Average Relation Neighbours Area	**57**	**Average Betweenness Centrality of slow cells**
**24**	S. D. Relation Neighbours Area	**58**	**S. D.** **Betweenness Centrality of slow cells**
**25**	Average Relation Neighbours major axis	59	Average Shortest Paths lengths
**26**	S. D. Relation Neighbours major axis	60	S. D. Shortest Paths Lengths
**27**	Average Relation Neighbours minor axis	**61**	**Average** **Shortest Paths Lengths from fast cells to fast cells**
**28**	S. D. Relation Neighbours minor axis	**62**	**S. D.** **Shortest Paths Lengths from fast cells to fast cells**
**29**	Average Relation Neighbours relation axis	**63**	**Average** **Shortest Paths Lengths from fast cells to slow cells**
**30**	S. D. Relation Neighbours relation axis	**64**	**S. D.** **Shortest Paths Lengths from fast cells to slow cells**
**31**	Average Relation Neighbours convex hull	**65**	**Average** **Shortest Paths Lengths from slow cells to slow cells**
**32**	S. D. Relation Neighbours convex hull	**66**	**S. D.** **Shortest Paths Lengths from slow cells to slow cells**
**33**	Average Relation Neighbours relation A1/A2	**67**	**Average** **Shortest Paths Lengths from slow cells to fast cells**
**34**	S. D. Relation Neighbours relation A1/A2	**68**	**S. D.** **Shortest Paths Lengths from slow cells to fast cells**
		**69**	**Proportion of slow cells**

Table shows the name of the 69 characteristics analyzed in the study. These characteristics can be classified into three types: geometrical characteristics, related to the size and shape of cells (1-14), network characteristics, capturing the organization of the cells (15-68) and the proportion of slow cells (69). The characteristics labelled in bold are the 35 features related to the fast or slow cell type. S. D. = Standard Deviation.

**Table 2:** Frequency of characteristics that better differentiate BA and QA images.

Name	Characteristic	Frequency
Feature selection: 1,000 combinations from BA (25 random images) vs BA (25 random images)		
S. D. Shortest Paths Lengths from fast cells to slow cells	64	20.60%
Average Shortest Paths Lengths from slow cells to slow cells	65	19.60%
Average Neighbours	15	16.60%
S. D. Shortest Paths Lengths from slow cells to fast cells	68	15.30%
S. D. Betweenness Centrality of slow cells	58	15%
Feature selection: 1,000 combinations from BA (25 random images) vs QA		
S. D. Area of slow cells	4	67.20%
Slow Neighbours of fast cells	21	48.40%
Average Strengths of fast cells	37	40.20%
Average Strengths of slow cells	39	36.80%
Average Area of fast cells	5	33.80%
S. D. Neighbours of slow cells	17	28.40%

This table shows the characteristics that have been selected with a higher frequency in the 1,000 BA-BA and BA-QA comparisons (using 69 characteristics).

**Table 3:** Comparison of real values and random values for each characteristic and type of muscle.

Biceps brachii Adult		Original	Random slow/fast cells		
Number	Characteristic	Value	Min	Max	Median
3	**Average Area of slow cells**	**23616.36**	25488.99	26776.38	26080.13
4	**S. D. Area of slow cells**	**4242.18**	5951.76	6964.85	6403.99
5	**Average Area of fast cells**	**27392.63**	25643.48	26536.55	26076.49
**6**	S. D. Area of fast cells	6409.93	6167.93	6785.72	6495.91
17	**S. D. Neighbours of slow cells**	**0.78**	0.79	0.90	0.84
**18**	S. D. Neighbours of fast cells	0.85	0.80	0.89	0.85
19	**slow Neighbours of slow cells**	**1.65**	2.56	2.82	2.69
20	**fast Neighbours of slow cells**	**4.26**	3.19	3.43	3.32
21	**slow Neighbours of fast cells**	**1.95**	2.64	2.84	2.74
22	**fast Neighbours of fast cells**	**4.14**	3.15	3.36	3.26
37	**Average Strengths of fast cells**	**1197.19**	1152.29	1183.17	1167.90
**38**	S. D. Strengths of fast cells	249.06	235.00	261.19	247.81
39	**Average Strengths of slow cells**	**1123.39**	1145.36	1189.76	1167.80
40	**S. D. Strengths of slow cells**	**221.58**	228.91	266.04	245.73
43	**Average Clustering Coefficient of fast cells**	**69.49**	69.70	71.68	70.73
**44**	S. D. Clustering Coefficient of fast cells	17.55	16.66	18.48	17.62
**45**	Average Clustering Coefficient of slow cells	72.27	69.13	72.45	70.73
**46**	S. D. Clustering Coefficient of slow cells	16.38	16.11	19.03	17.43
**49**	Average Eccentricity of fast cells	2102.00	2079.41	2112.54	2095.04
**50**	S. D. Eccentricity of fast cells	269.37	254.36	276.71	264.78
**51**	Average Eccentricity of slow cells	2080.82	2074.78	2117.68	2095.31
**52**	S. D. Eccentricity of slow cells	250.47	248.99	277.91	263.52
**55**	Average Betweenness Centrality of fast cells	1324.80	1221.65	1438.54	1314.93
**56**	S. D. Betweenness Centrality of fast cells	1004.46	791.91	1195.70	965.58
**57**	Average Betweenness Centrality of slow cells	1330.29	1227.92	1415.92	1316.04
**58**	S. D. Betweenness Centrality of slow cells	971.95	801.17	1140.23	978.55
**61**	Average Shortest Paths Lengths from fast cells to fast cells	683.97	662.75	693.15	676.53
**62**	S. D. Shortest Paths Lengths from fast cells to fast cells	309.38	299.37	312.65	305.66
63	**Average Shortest Paths Lengths from fast cells to slow cells**	**789.92**	707.30	772.82	739.85
64	**S. D. Shortest Paths Lengths from fast cells to slow cells**	**273.72**	277.99	301.80	288.77
**65**	Average Shortest Paths Lengths from slow cells to slow cells	663.20	653.53	697.28	676.70
**66**	**S. D. Shortest Paths Lengths from slow cells to slow cells**	**290.31**	291.45	313.76	300.95
**67**	**Average Shortest Paths Lengths from slow cells to fast cells**	**645.91**	682.38	724.70	703.57
**68**	S. D. Shortest Paths Lengths from slow cells to fast cells	301.25	285.64	302.72	294.66
**Quadriceps Adult**		**Original**	**Random slow/fast cells**		
**Number**	**Characteristic**	**Value**	**Min**	**Max**	**Median**
**3**	Average Area of slow cells	17252.13	15902.67	17518.80	16705.52
**4**	S. D. Area of slow cells	4522.11	4492.86	5516.13	4985.71
5	**Average Area of fast cells**	**16122.16**	16396.41	16980.73	16705.20
6	**S. D. Area of fast cells**	**4617.04**	4829.16	5257.62	5054.59
**17**	S. D. Neighbours of slow cells	0.88	0.82	1.03	0.92
**18**	S. D. Neighbours of fast cells	0.92	0.89	0.95	0.93
**19**	slow Neighbours of slow cells	1.44	1.28	1.64	1.46
**20**	fast Neighbours of slow cells	4.48	4.33	4.76	4.55
**21**	slow Neighbours of fast cells	1.51	1.41	1.57	1.50
**22**	fast Neighbours of fast cells	4.49	4.40	4.61	4.51
**37**	Average Strengths of fast cells	934.22	928.50	951.11	939.90
**38**	S. D. Strengths of fast cells	216.79	210.15	226.43	218.86
**39**	Average Strengths of slow cells	930.21	908.02	974.15	939.80
**40**	S. D. Strengths of slow cells	200.14	188.05	240.27	214.84
**43**	Average Clustering Coefficient of fast cells	58.22	57.36	58.93	58.20
**44**	S. D. Clustering Coefficient of fast cells	14.64	13.90	15.23	14.64
**45**	Average Clustering Coefficient of slow cells	58.58	55.97	60.57	58.20
**46**	S. D. Clustering Coefficient of slow cells	14.33	12.48	16.29	14.36
**49**	Average Eccentricity of fast cells	1968.09	1953.97	1976.74	1965.88
**50**	S. D. Eccentricity of fast cells	255.27	244.85	260.67	252.97
**51**	Average Eccentricity of slow cells	1959.02	1924.17	2004.91	1966.09
**52**	S. D. Eccentricity of slow cells	246.90	220.88	279.75	250.01
**55**	Average Betweenness Centrality of fast cells	2039.74	1929.36	2108.39	2024.31
**56**	S. D. Betweenness Centrality of fast cells	1508.94	1256.21	1593.69	1474.73
**57**	Average Betweenness Centrality of slow cells	1934.73	1770.01	2334.29	2020.35
**58**	S. D. Betweenness Centrality of slow cells	1222.43	1020.43	1850.30	1410.62
**61**	Average Shortest Paths Lengths from fast cells to fast cells	649.27	641.89	661.91	652.31
**62**	S. D. Shortest Paths Lengths from fast cells to fast cells	299.85	295.33	304.30	300.17
**63**	Average Shortest Paths Lengths from fast cells to slow cells	823.40	764.99	863.39	819.00
**64**	S. D. Shortest Paths Lengths from fast cells to slow cells	274.52	252.49	293.78	274.44
**65**	Average Shortest Paths Lengths from slow cells to slow cells	664.56	615.26	692.43	652.40
**66**	S. D. Shortest Paths Lengths from slow cells to slow cells	296.06	279.01	315.80	295.42
**67**	Average Shortest Paths Lengths from slow cells to fast cells	625.33	603.43	639.35	619.50

The table shows results of the evaluation of the 34 characteristics related to the fast and slow condition of the fibres. Each original value is compared with the minimum, maximum and median values for 10,000 randomizations. The original values labelled in bold mark the ones outside of the range of values of the random distribution in each case.
