# Supplementary material for: Rules of tissue packing involving different cell types: human muscle organization
Source: Sci Rep. 2017 Jan 10;7:40444. doi: 10.1038/srep40444 (PMC5223128; doi:10.1038/srep40444)
Supplement: Supplementary Information [file srep40444-s1.pdf]

Rules of tissue packing involving different cell types: human muscle organization

Daniel Sánchez-Gutiérrez, Aurora Sáez, Pedro Gómez-Gálvez, Carmen Paradas, Luis M. Escudero.

SUPPLEMENTARY INFORMATION

Table S1 related to Table 1

Values for the 69 characteristics extracted form the 90 BA ROIs and the 25 QA ROIs.

Table S2 related to Table 1

Average value of each characteristic for BA and QA samples. The last column shows the *p value* for the Student's t-test comparing the average values of each characteristic. In red are labelled the *p values* that are lower than 0.05.

Table S3 related to Table 2

The tables show all the characteristics that appear in more than a 15% of the cases in different BA-BA and BA-QA comparisons.

Table S4 related to Figure 4

Statistical tests related to **Figure 4**. The upper table shows the MANOVA *p values* for the comparison of the total polygon distribution. The lower table shows Student's t-test (green) and Wilcoxon (blue) *p values* for the comparisons of the distributions for each type of polygon (4, 5, 6, 7 and 8 sides).

Figure S1 related to Figure 2

PCA graphs for the comparisons between total BA and QA samples using different sets of characteristics. The green dots represent BA images and the red dots QA images. The numbers over the graphs indicate the selected characteristics. **A)** 90 samples of BA versus 25 QA images using the set of 69 cc. **B)** 90 images of BA versus 25 QA images using the set of 15 cc. **C)** 90 images of BA versus 25 QA images using the set of 35 cc.

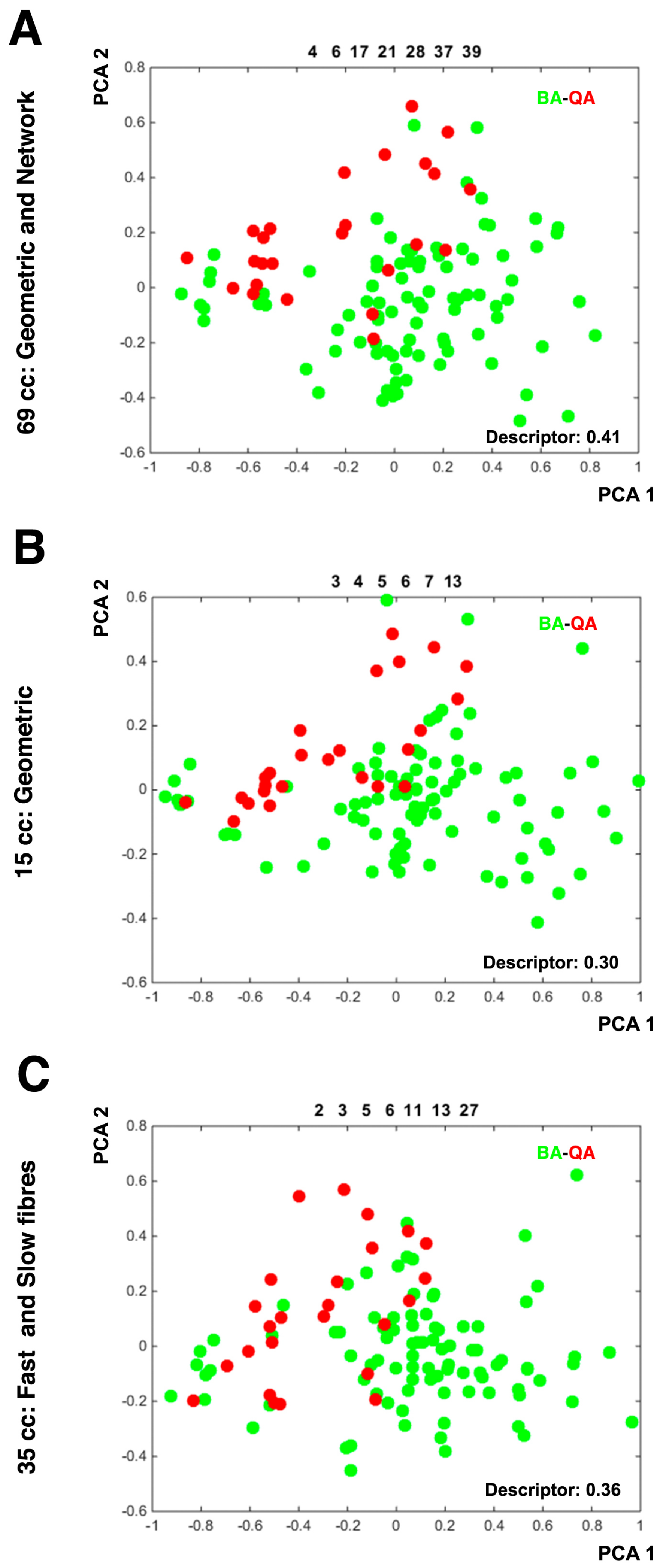
A
69 cc: Geometric and Network
4 6 17 21 28 37 39
BA-QA
Descriptor: 0.41
PCA 1
PCA 2
B
15 cc: Geometric
3 4 5 6 7 13
BA-QA
Descriptor: 0.30
PCA 1
PCA 2
C
35 cc: Fast and Slow fibres
2 3 5 6 11 13 27
BA-QA
Descriptor: 0.36
PCA 1
PCA 2
